# Interdependence of Bad and Puma during Ionizing-Radiation-Induced Apoptosis

**DOI:** 10.1371/journal.pone.0088151

**Published:** 2014-02-06

**Authors:** Cristhian Toruno, Seth Carbonneau, Rodney A. Stewart, Cicely Jette

**Affiliations:** 1 Department of Oncological Sciences, Huntsman Cancer Institute, University of Utah, Salt Lake City, Utah, United States of America; 2 Department of Pediatric Oncology, Dana-Farber Cancer Institute and Harvard Medical School, Boston, Massachusetts, United States of America; Innsbruck Medical University, Austria

## Abstract

Ionizing radiation (IR)-induced DNA double-strand breaks trigger an extensive cellular signaling response that involves the coordination of hundreds of proteins to regulate DNA repair, cell cycle arrest and apoptotic pathways. The cellular outcome often depends on the level of DNA damage as well as the particular cell type. Proliferating zebrafish embryonic neurons are highly sensitive to IR-induced apoptosis, and both p53 and its transcriptional target *puma* are essential mediators of the response. The BH3-only protein Puma has previously been reported to activate mitochondrial apoptosis through direct interaction with the pro-apoptotic Bcl-2 family proteins Bax and Bak, thus constituting the role of an “activator” BH3-only protein. This distinguishes it from BH3-only proteins like Bad that are thought to indirectly promote apoptosis through binding to anti-apoptotic Bcl-2 family members, thereby preventing the sequestration of activator BH3-only proteins and allowing them to directly interact with and activate Bax and Bak. We have shown previously that overexpression of the BH3-only protein Bad in zebrafish embryos supports normal embryonic development but greatly sensitizes developing neurons to IR-induced apoptosis. While Bad has previously been shown to play only a minor role in promoting IR-induced apoptosis of T cells in mice, we demonstrate that Bad is essential for robust IR-induced apoptosis in zebrafish embryonic neural tissue. Moreover, we found that both p53 and Puma are required for Bad-mediated radiosensitization *in vivo*. Our findings show the existence of a hierarchical interdependence between Bad and Puma whereby Bad functions as an essential sensitizer and Puma as an essential activator of IR-induced mitochondrial apoptosis specifically in embryonic neural tissue.

## Introduction

Cellular exposure to ionizing radiation (IR) causes multiple forms of DNA damage, including single- and double-stranded breaks (DSB), within the sugar-phosphate backbone of DNA. When two single-stranded breaks occur in close proximity to each other, the resultant DSB represents a threat to genome stability. Thus, the presence of DNA DSBs elicits an extensive cellular response that coordinates decisions to undergo cell cycle arrest, DNA repair and apoptosis. In response to IR exposure, certain cell types appear to be programmed to favor cell cycle arrest and DNA repair over apoptosis (e.g. fibroblasts) and vice versa (e.g. T cells) [1]. Moreover, the sensitivity of tissues to IR-induced apoptosis can change over the course of development. For instance, proliferating embryonic neurons are highly sensitive to IR-induced apoptosis whereas differentiated, mature neurons are highly resistant [1], [2].

The DSB-DNA damage response (DSB-DDR) pathway involves the transcriptional and post-translational regulation of hundreds of genes [3], [4]. Critical mediators of the response include the Atm and Atr kinases, which phosphorylate and activate the checkpoint kinases, Chk2 and Chk1, respectively. These post-translational mechanisms induce rapid cell cycle arrest at G1/S, intra-S, and G2 phases of the cell cycle. DNA repair mechanisms then attempt to repair the DNA before resuming progression through the cell cycle. Phosphorylation and activation of the transcription factor p53 by Atm and Chk2 can also stabilize the G1/S phase checkpoint through induction of the cell cycle inhibitor *p21* and can induce apoptosis through induction of pro-apoptotic BH3-only genes like *puma* and *noxa*. Due to the requirement for transcription, p53-mediated cell cycle arrest and apoptosis represent a delayed response to IR [3], [4].

IR-mediated apoptosis occurs through engagement of the intrinsic, mitochondrial pathway. Members of the Bcl-2 family of proteins are critical mediators of mitochondrial apoptosis [5], [6], [7]. All members of this family have been identified through conservation of one or more of the four alpha-helical domains that reside within the amino acid sequence of the founding member Bcl-2, called Bcl-2 homology (BH) domains. Most anti-apoptotic members include all four BH domains (Bcl-2, Bcl-xL, Mcl-1, Bcl-w and Bfl-1). Pro-apoptotic multidomain members Bax and Bak show conservation of BH domains 1-3, and pro-apoptotic BH3-only proteins (Bim, Bid, Bad, Bik, Puma, Noxa, Bmf, BNIP3, Bmf, Hrk, MULE) show conservation of only the BH3 domain. Transcriptional induction or post-translational activation of BH3-only proteins leads to homo-oligomerization and activation of Bax and Bak either by direct binding, or indirectly through inactivation of the anti-apoptotic Bcl-2 family members. Homo-oligomerization of Bax and/or Bak is thought to create pores in the mitochondria that allow release of cytochrome C. In the cytoplasm, cytochrome C can pair with Apaf-1 and the initiator procaspase-9 to form the “apoptosome” which initiates a caspase cascade through activation of effector Caspases 3, 6 and 7. This culminates in the proteolytic destruction of the cell and the subsequent hallmarks of apoptosis, such as membrane blebbing, DNA condensation and fragmentation, and cell surface signals that mediate packaging of the cell for engulfment by phagocytes in an immune silent manner [5], [6], [7].

BH3-only proteins can be classified as either activators or sensitizers, based on the mechanism by which they cause activation of Bax and Bak [5], [8]. Activators, like Bid, Bim and Puma, can bind directly to Bax and Bak to induce their homo-oligomerization. Sensitizers comprise the remaining BH3-only proteins and activate Bax and Bak indirectly by binding to the anti-apoptotic Bcl-2 family members. This mediates the release of activators from anti-apoptotic members, allowing the activators to directly bind and activate Bax and Bak. This establishes a hierarchy among the BH3-only proteins in that sensitizers act genetically upstream of activators [5], [8].

The p53 transcriptional targets *puma* and *noxa* mediate p53’s proapoptotic function in response to the induction of DNA-DSBs [9]. Puma has been shown to be critical for DSB-DNA-damage-induced apoptosis while Noxa’s role tends to be more modest and restricted to cell type. Bad sensitizes thymocytes exclusively to IR whereas it sensitizes mouse embryonic fibroblasts to a number of different apoptotic stimuli [10]. In 24-hpf zebrafish embryonic neurons, p53 and Puma (but not Noxa), are absolutely required for IR-induced apoptosis [11], [12].

Overexpression of *puma* in zebrafish embryos leads to massive apoptosis and rapid death of the embryo whereas embryos injected with *bad* mRNA develop normally [12], [13]. However, at 24 hours-post-fertilization (hpf), Bad-expressing embryos are markedly sensitized to IR-induced apoptosis suggesting that IR induces the pro-apoptotic activity of Bad [13]. Mammalian BAD pro-apoptotic activity is well-known to be regulated by phosphoregulation of critical serines in response to growth factor withdrawal [14]. Serines 112, 136 and 155 (mouse BAD_L_ enumeration) have been shown to be critical in the context-dependent regulation of BAD activity by several kinases and phosphatases. Both PKA and p90RSK phosphorylate serines 112 and 155 [15], [16], [17] while AKT and p70S6 kinases target S136 [18], [19], [20], [21], [22]. The phosphatases Calcineurin, PP2A and PP1 have also been shown to dephosphorylate these critical serines within specific death paradigms [23], [24], [25], [26], [27], [28], [29]. Serine 155 lies in the middle of the BH3 domain of BAD and when phosphorylated, precludes binding to BCL-2 and BCL-xL, thus rendering BAD apoptotically inactive [30], [31], [32]. However, regulation of the three serines seems to occur in a tiered fashion in that the initial phosphorylation of S112 and S136 (and their subsequent binding to 14-3-3 proteins) may be required to permit the accessibility of S155 kinases to the BH3 domain [15]. Regulation of S112 is not likely to be required for the ability of Bad to radiosensitize zebrafish embryos since it does not appear to be conserved in the zebrafish Bad protein [13]. However, we have previously shown that phosphorylation of zBad serines 84 and 103 (that show conservation with mouse BAD serines 136 and 155, respectively) likely inhibits Bad’s pro-apoptotic activity until IR causes serine dephosphorylation and activation of Bad [13].

Here we investigate the mechanisms by which Bad sensitizes zebrafish neural tissue to IR. We find that Bad-mediated radiosensitization requires p53 but does not influence its transcriptional activity. The p53 target Puma is also required for Bad-mediated radiosensitization. Surprisingly, we show that similar to Puma, endogenous Bad is essential for robust IR-induced apoptosis. These non-redundant functions for Bad and Puma suggest a model whereby Puma acts as an essential activator BH3-only protein that requires the sensitizer BH3-only protein Bad to promote IR-induced mitochondrial apoptosis.

## Materials and Methods

### Ethics Statement

All experiments involving zebrafish were carried out in strict accordance with the recommendations in the Guide for the Care and Use of Laboratory Animals of the National Institutes of Health. The protocol was approved by the University of Utah Institutional Animal Care and Use Committee (Protocol numbers: 10-02003 and 13-01003), and all efforts were made to minimize suffering.

### Zebrafish Lines

Zebrafish were maintained and bred as described [33]. Wild-type embryos were derived from the AB strain. The *p53^e7/e7^* zebrafish line that carries a homozygous missense mutation encoding a M214K substitution in the p53 protein has been previously described [11].

### Irradiator Usage

IR was administered with either a Cs-137 gamma irradiator (Gammacell 1000) or an X-ray irradiator (RadSource RS2000). Irradiators were completely interchangeable such that 8 Gy X-irradiation gave rise to identical embryonic phenotypes as 8 Gy gamma-irradiation.

### Zebrafish Microinjections

Zebrafish one-cell stage embryos were injected with the indicated amounts of mRNA or morpholino. In every experiment, total RNA or morpholino concentrations were kept constant through the use of *mcherry*/*egfp* mRNA or a mismatch morpholino, respectively. Morpholinos were designed and created by GeneTools Inc. For mRNA microinjection, zebrafish cDNAs were sub-cloned into pCS2+, and mRNA was made by 1) linearization of each construct with NotI, 2) SP6 Message Machine kit (Ambion, AM1340) and 3) purification for microinjection with NucAway Spin Columns (Ambion, AM10070). Sequences of morpholinos and primers are listed in Table S1.

### Whole-mount Immunofluorescence and Quantitation

Whole-mount activated Caspase 3 immunofluorescence (the Casp3 assay) was performed and quantified as described previously [34], [35]. At least ten embryos from each group were included for all quantifications. GraphPad Prism software was used to plot the data, and error bars represent the standard error of averaged data from the embryos in a single experiment (in some experiments, as indicated, data from multiple experiments was averaged). Statistical analyses were performed in GraphPad Prism using an unpaired student’s T test. Quantification represents measurements of fluorescence intensity that are directly related to Caspase 3 activity. However, fluorescence intensity is likely to fluctuate within cells. Therefore, changes in fluorescence intensity likely represent both increasing apoptotic cell number as well as increasing Caspase 3 activity within individual cells.

### RT-PCR and Quantitative Real-time PCR

RNA was isolated from embryos (at least 20 embryos/sample) using the Qiagen RNeasy kit (74104). One microgram of purified RNA was used to generate cDNA using the Invitrogen Thermoscript RT-PCR kit (11146-024) and oligo-dT primers. cDNA was diluted 1:20 in nuclease-free water (Ambion). For analysis of the *bad e2i2* morpholino, RT-PCR was performed and analyzed by standard agarose gel electrophoresis. For quantitative real-time PCR, three technical replicates were analyzed using an Eppendorf Realplex system. Primers were designed by Roche to be used with the Universal Probe Library. All primers used for RT-PCR and qPCR are listed in Table S1. GraphPad Prism software was used to plot the data, and error bars represent the standard error of averaged data. Statistical analyses were performed in GraphPad Prism using an unpaired student’s T test.

## Results

### Bad is Required for IR-induced Apoptosis in Zebrafish Neural Tissue

We previously showed that overexpression of zebrafish Bad sensitizes zebrafish neural tissue to IR-induced apoptosis [13]. To investigate conservation of function between zebrafish and human Bad proteins, we compared the radiosensitivity induced by overexpression of zebrafish Bad and human BAD. We injected one-cell stage embryos with 50 pg of mRNA encoding *BAD* (or *mcherry* as a control), exposed half of each group to 8 Gy IR at 24 hours post fertilization (hpf), and analyzed apoptosis three hours later by immunofluorescence to detect activated Caspase 3 (hereafter referred to as the Casp3 assay). Fluorescence intensity in the spinal cords of zebrafish embryos was analyzed to compare levels of IR-induced apoptosis, as previously described [13], [35]. Exposure of zebrafish embryos to 8 Gy elicits a quantitatively moderate apoptotic response to IR such that genetic manipulations that sensitize the embryo to IR can be identified and measured [13], [34], [35]. Similar to zBad [13], hBAD radiosensitizes zebrafish neural tissue approximately 8-fold (Figure S1).

**Figure 1:**
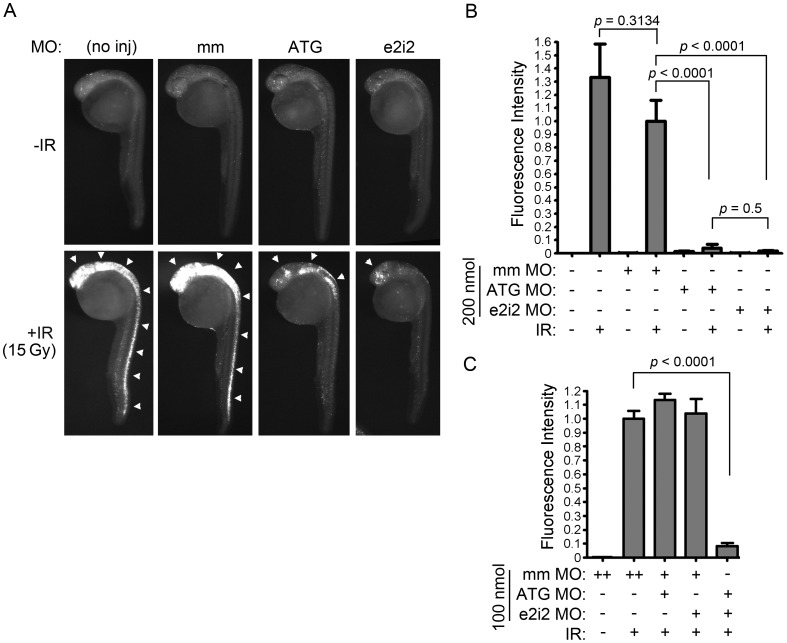
Bad is required for IR-induced apoptosis in zebrafish embryonic neural tissue. (A) Shown are lateral views of 27-hpf embryos (head is top left in each panel) either uninjected or injected with 200 nmol of *bad ATG*, *bad e2i2* or mismatch (mm) MO. Half of each group of embryos were exposed to 15 Gy IR, and all were analyzed by the Casp3 assay. In control embryos (no inj and mm), IR-induced apoptosis occurs predominantly in the brain and all along the spinal cord (white arrowheads), whereas in *bad*-deficient embryos (ATG and e2i2), residual apoptosis is only observed in the head (arrowheads). (B) Fluorescence intensity, reflecting level of Caspase 3 activity, was measured in the spinal cords of at least 10 embryos from each group in (A) as previously described [34]. The fluorescence intensity in irradiated mismatch-MO-injected embryos was normalized to 1. (C) One-cell stage zebrafish embryos were injected with 100 nmol of *bad ATG*, *bad e2i2* or mm MO as indicated (“++” indicates that 200 nmol was injected to keep total concentration of MO constant between experimental groups) and irradiated and analyzed as in (A-B). Data represent one experiment, and the experiment was independently performed three times with similar results.

To determine whether endogenous Bad normally promotes IR-induced apoptosis in this model system, we generated both a translation-blocking (*bad-ATG-MO*) and splice-blocking (*bad-e2i2-MO*) morpholino (MO, Figure S2) to test whether knockdown of zebrafish Bad expression inhibits the apoptotic response to IR. One-cell stage wild-type embryos were injected with 200 nmol of either MO targeting *bad*, or a mismatch control MO. At 24 hpf, embryos were exposed to 15 Gy IR [which produces a quantitatively maximal apoptotic response in zebrafish neural tissue (data not shown) such that genetic manipulations that protect the embryo from IR can be identified and measured] and analyzed three hours later by the Casp3 assay. Figures 1A-B show that both MOs severely impair the ability of IR to induce apoptosis in zebrafish neural tissue. The splice-blocking MO leads to the inclusion of intron 2 and inhibits Bad activity by incorporating an early stop codon in the *bad* transcript (Figure S2). To prove that the MOs were specifically targeting *bad*, we attempted to rescue the radioprotective phenotype by overexpressing *hBAD* or *zbad* mRNA in combination with each morpholino. However, the combination of each *bad* MO plus the *bad* mRNA proved highly pro-apoptotic to the developing embryos and precluded the analysis of IR-specific apoptosis. This is possibly due to the ability of high levels of *bad* mRNA to reveal a non-specific toxicity of these particular morpholinos, an effect we have seen with *bad* mRNA in other contexts (data not shown). To evaluate the specificity of the MOs using a different approach, we analyzed the effect of combining suboptimal concentrations of both MOs. Figure 1C shows that injection of 100 pmol of either MO fails to inhibit IR-induced apoptosis while their combination severely impairs the apoptotic response. Thus, Bad is a critical mediator of IR-induced apoptosis in zebrafish developing neural tissue.

### p53 is Required for Bad-mediated Sensitization to IR but not to Wortmannin

**Figure 2:**
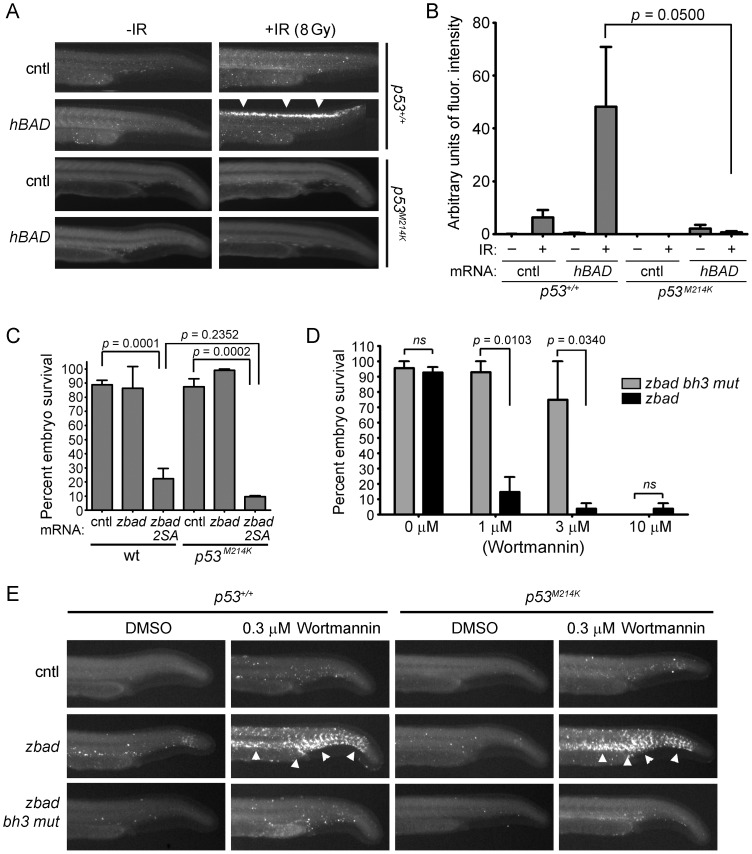
p53 is required for Bad-mediated sensitivity to IR but not wortmannin. (A) Shown are lateral views of representative tails from 27-hpf wild-type or *p53* mutant embryos injected with 50 pg of *mcherry* (cntl) or *hBAD* mRNA. Embryos were exposed (or not) to 8 Gy IR at 24 hpf and analyzed three hours later by the Casp3 assay. Apoptosis was observed in the spinal cord after *hBAD* mRNA was injected into wild-type (arrowheads), but not mutant, *p53* embryos. (B) Fluorescence intensity was measured in the spinal cords of at least 10 embryos from each group in (A). Data represent one experiment, but the experiment was independently performed three times with similar results. (C) One-cell stage wild-type or *p53* mutant embryos were injected with 50 pg of mRNA encoding either *mcherry* control (cntl) or the constitutively active mutant *zbad 2SA*. At 8 hpf, embryos were analyzed for survival (defined by a beating heart) as performed previously [34]. Data represent one experiment, but the experiment was independently performed three times with similar results. (D) One-cell stage wild-type embryos were injected with 50 pg of mRNA encoding either zebrafish *bad* or the apoptotically-inactive *zbad bh3 mut*. At 8 hpf, embryos were treated with increasing concentrations of wortmannin, or DMSO vehicle alone. At 48 hpf, embryos were examined for survival. At least 10 embryos were analyzed per group in three independent experiments. E) Shown are lateral views of tails from *p53* wild-type (left) or mutant (right) embryos after injection with 25 pg of mRNA encoding either *mcherry* (cntl), *zbad*, or *zbad bh3 mut*. Embryos were split into two groups and treated with either 0.3 µM wortmannin or DMSO vehicle beginning at 8 hpf and analyzed at 24 hpf by the Casp3 assay. Wild-type Bad synergizes with wortmannin to induce apoptosis in multiple tissues in a *p53*-independent manner (arrowheads).

The tumor suppressor p53 is a critical component of the DSB-DDR pathway [1] and is absolutely required for IR-induced apoptosis in zebrafish neural tissue [11]. To determine whether p53 is also required for Bad-mediated radiosensitization, we analyzed the effect of Bad overexpression on IR-induced apoptosis in both wild-type and transcriptionally-inactive *p53* mutant embryos (*p53^M214K^,* [11]). We injected one-cell stage embryos (either wild-type or *p53* mutant) with 50 pg of *BAD* (or control) mRNA, exposed half the embryos to 8 Gy IR at 24 hpf, and analyzed apoptosis three hours later by the Casp3 assay. Embryo tails (rather than the whole embryo) are displayed in this figure and for subsequent experiments in this study to enhance the reader’s ability to visually analyze apoptosis and to remind the reader that Caspase 3 activity was measured in the spinal cords of embryo tails. Figures 2A-B demonstrate that *p53* is required for Bad-mediated radiosensitization of zebrafish neural tissue. However, these experiments do not distinguish whether p53 is required upstream (e.g. for the activation of Bad) or downstream (e.g. for active Bad to induce apoptosis) of Bad activity. To investigate these two possibilities, we asked whether wild-type *p53* is required for the pro-apoptotic activity of a constitutively active Bad mutant in which serines 84 and 103 have been mutated to alanine (referred to as Bad 2SA). Our previous work showed that injection of zebrafish embryos with mRNA encoding *bad 2SA* leads to massive apoptosis followed by death of most embryos by 8 hours-post-injection [13]. We therefore performed the same experiment by injecting mRNA encoding either *bad 2SA*, or *mcherry* as a control, into either wild-type or *p53* mutant embryos and analyzed embryonic death at 8 hpf. Figure 2C shows that *p53* is not required for active Bad to induce apoptosis.

We next questioned whether p53 is required for the activation of Bad by an IR-independent stimulus. To establish an IR-independent stimulus of Bad in zebrafish embryos, we took advantage of the previous finding that Akt can inhibit the pro-apoptotic function of Bad by phosphorylating Serine 136 on human BAD [18], [19], [20], [21]. We therefore reasoned that inhibition of the PI3K/Akt pathway in developing zebrafish embryos would elicit an apoptotic response in combination with Bad overexpression. To test this, we injected one-cell stage embryos with 50 pg of mRNA encoding either *bad* or *bad bh3 mut,* which contains a leucine to alanine mutation in the first amino acid of the BH3 domain of Bad (L99A) and completely inhibits its pro-apoptotic activity [13]. At 8 hpf, we treated embryos in their chorions with increasing doses of the PI3K inhibitor wortmannin. We then let the embryos develop until 48 hpf and analyzed their morphology and survival. We found that in the absence of wortmannin, nearly all embryos developed normally (Figure 2D, S3). At 1 and 3 µM wortmannin, however, most *bad*-expressing embryos showed signs of severe developmental defects that culminated in embryonic death while most *bad bh3 mut*-expressing embryos developed normally. At 10 µM wortmannin, both *bad*- and *bad bh3 mut*-expressing embryos succumbed to embryonic death suggesting that this concentration of wortmannin has toxic effects on the embryos that are independent of Bad overexpression (but are perhaps due to synergy with endogenous Bad). These data indicate that 3 µM or lower concentrations of wortmannin specifically synergize with the pro-apoptotic activity of overexpressed Bad to induce embryonic cell death.

Since we established wortmannin treatment as an IR-independent stimulus of Bad’s pro-apoptotic activity, we next asked whether p53 is required for the synergy between wortmannin and Bad. We wished to specifically analyze apoptosis in the absence of severe morphological defects, so we sought to examine an interaction between suboptimal doses of wortmannin and Bad that supported nearly normal development. To achieve this, one-cell stage wild-type or *p53* mutant embryos were injected with 25 pg of mRNA encoding *bad*, or *bad bh3 mut* or *mcherry* as controls. At 8 hpf, embryos were treated with 0.3 µM wortmannin and analyzed at 24 hpf by the Casp3 assay. Representative embryo tails in Figure 2E show that Bad and wortmannin synergize to induce apoptosis in multiple embryonic tissues in a manner that is dependent on Bad’s pro-apoptotic activity. Importantly, wild-type p53 is not required for this synergy. These experiments indicate that wortmannin causes activation of Bad, likely through serine dephosphorylation, and that the activation of Bad is not dependent on p53.

Overall, these experiments indicate that p53 is not inherently required for either the activation of Bad, or for active Bad to induce apoptosis. Thus, we sought to further understand why p53 is specifically required for Bad to induce apoptosis in response to IR.

### Bad does not Alter the Timing of IR-induced Apoptosis

IR-induced apoptosis represents a delayed response to DNA damage since it requires time for p53-mediated transcription and translation of pro-apoptotic targets like *puma*. However, p53 has also been shown to regulate DNA repair and promote apoptosis by transcription-independent mechanisms [36], [37]. We therefore reasoned that if Bad requires p53-mediated transcription to radiosensitize zebrafish neural tissue, then Bad overexpression should not alter the timing of IR-induced apoptosis. To test this, we injected one-cell stage wild-type embryos with mRNA encoding either *BAD* or *mcherry*, irradiated half the embryos at 24 hpf, and analyzed apoptosis by the Casp3 assay at one, two, and three hours post-irradiation (hpIR). Figure S4 demonstrates that while overexpression of BAD enhances IR-induced apoptosis at two and three hpIR, it does not change the timing of apoptosis induction following IR. This result is consistent with a requirement for p53-mediated transcription during Bad-mediated radiosensitization.

### Bad Radiosensitizes in a Pathway that is either Downstream or Parallel to p53 Activity

**Figure 3:**
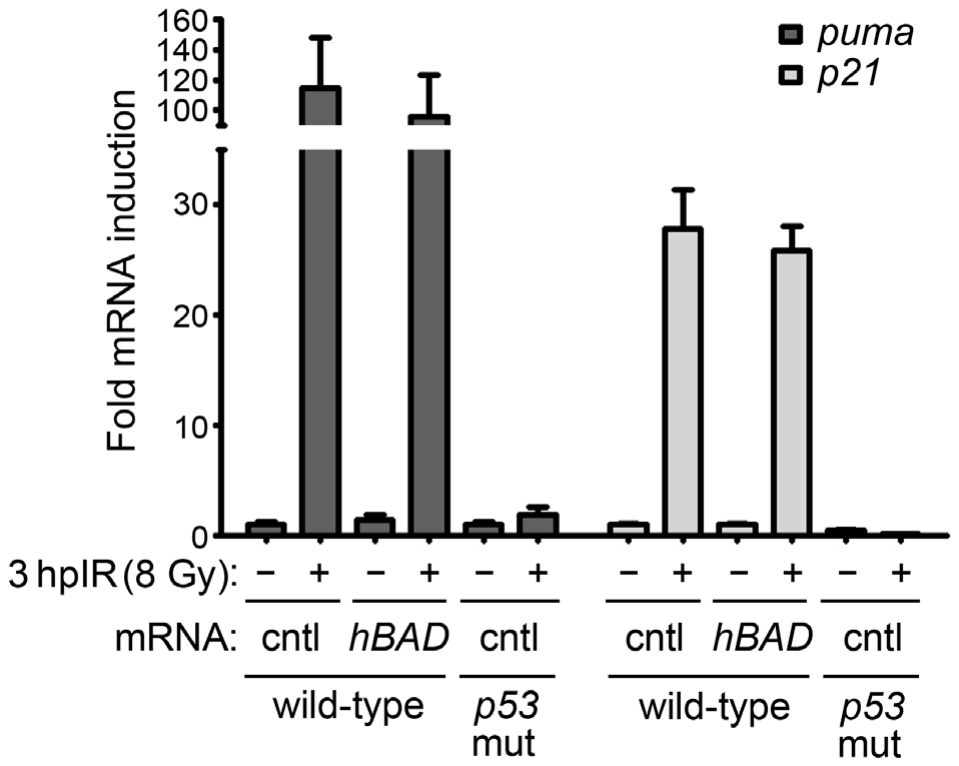
Bad does not augment p53 transcriptional activity. p53 transcriptional activity was analyzed in embryos injected with 50*mcherry* (cntl) or *hBAD*. Embryos were exposed to 8 Gy (or not) at 24 hpf. RNA was harvested from each group at 27 hpf and analyzed for gene expression changes by qPCR. Expression of the *gapdh* gene was measured to normalize *puma* and *p21* mRNA levels. All data was compared to unirradiated wild-type control-mRNA-injected data, which was adjusted to a value of 1. Control-injected *p53* mutant embryos irradiated at 24 hpf and harvested at 27 hpf were included as a negative control for p53-mediated transcriptional induction. Data represent one experiment, but the experiment was independently performed three times with similar results.

Bad is well-known to promote apoptosis through its function as a BH3-only protein. However, one simple explanation for the ability of Bad to augment IR-induced apoptosis in a p53-dependent manner is by increasing the activity of p53. To therefore determine whether Bad is causing an increase in p53 transcriptional activity, we tested the induction of the p53 transcriptional targets *puma* and *p21* in irradiated embryos with normal (endogenous) or high levels of Bad expression. We injected one-cell stage wild-type embryos with mRNA encoding either *mcherry* or *BAD,* irradiated half of each group at 24 hpf and analyzed all embryos at 3 hpIR by qPCR for *puma* and *p21* expression. Figure 3 shows that both *puma* and *p21* are induced by IR to a similar degree in both mcherry- and BAD-expressing embryos indicating that Bad does not influence p53 activity in order to radiosensitize embryos.

### Puma is Required for Bad-mediated Radiosensitization

**Figure 4:**
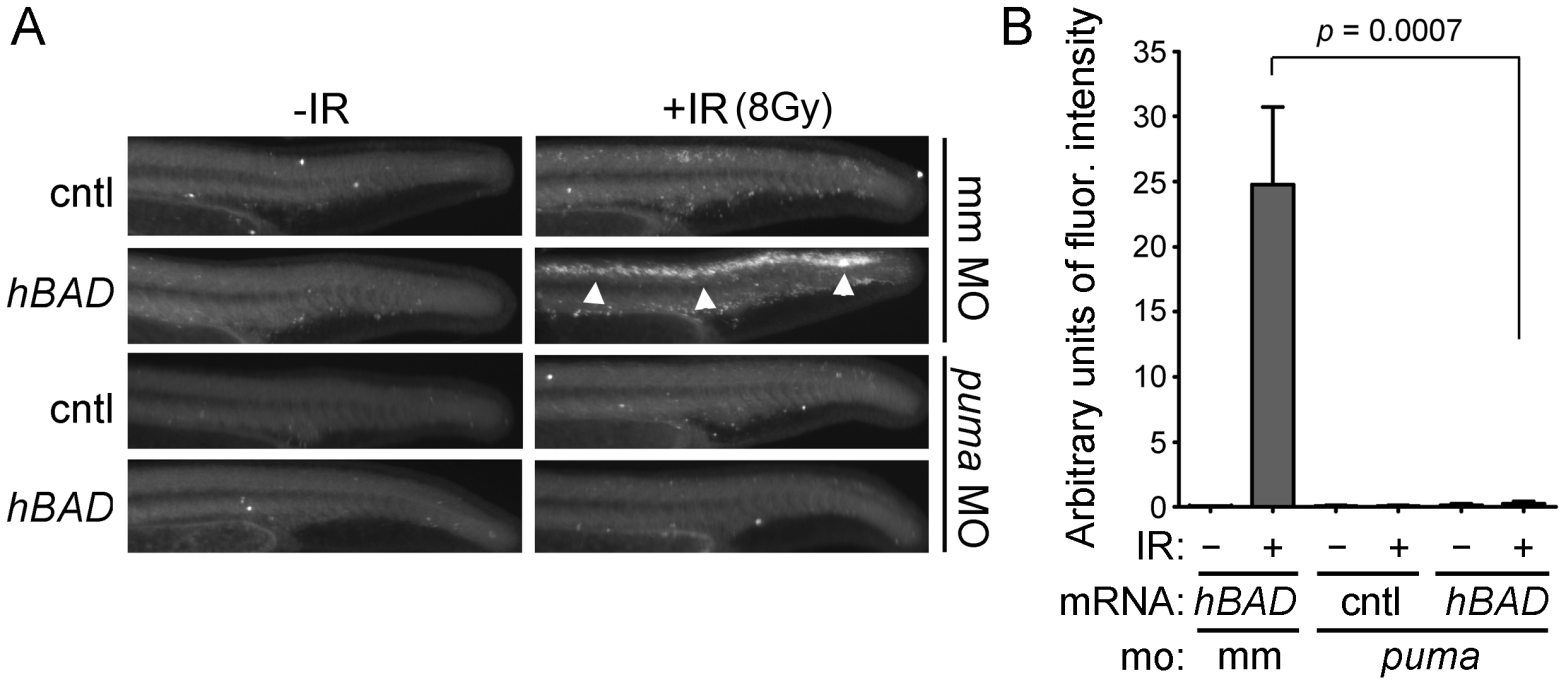
Puma is required for Bad-mediated radiosensitization. (A) Shown are lateral views of representative tails from 27-hpf embryos exposed to 8 Gy IR after injection at the one-cell stage with 50 pg of mRNA encoding *mcherry* (cntl) or *hBAD* in addition to either 100 nmol of *puma* MO or mismatch (mm) control MO. Analysis of Caspase 3 activity shows that *hBAD*-mediated radiosensitivity (arrowheads) is dependent on *puma* expression. (B) Fluorescence intensity was measured in the spinal cords of at least 10 embryos from each group in (A). Data represent one experiment, but the experiment was independently performed three times with similar results.

The p53 transcriptional target *puma* is a critical component of the DSB-DDR pathway [9]. Indeed, it has been shown to be absolutely required for IR-induced apoptosis in zebrafish neural tissue [12], [38]. Puma has been implicated as an activator BH3-only protein [39] while Bad appears to be a *bona fide* sensitizer [8], [40], [41]. This suggests that Puma could potentially function downstream of Bad during Bad-mediated radiosensitization. To test this possibility, we analyzed the effect of *BAD* mRNA overexpression on IR-induced apoptosis in *puma* morphants. We co-injected embryos with *BAD* mRNA in addition to 100 pg *puma* (or control) MO, irradiated half of each group at 24 hpf and analyzed them three hours later by the Casp3 assay. Figure 4A/B shows that *puma* is required for *BAD*-mediated radiosensitization of zebrafish neural tissue.

### Bcl-xL Expression Blocks Bad-mediated Radiosensitivity

**Figure 5:**
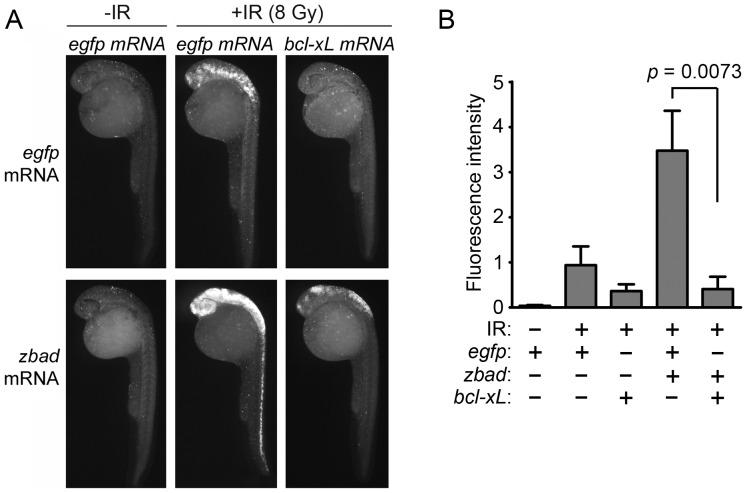
Bad radiosensitizes zebrafish neural tissue through the mitochondrial apoptosis pathway. (A) Shown are lateral views of 27-hpf wild-type embryos (head is top left in each panel) injected with either 1) 50 pg of *egfp* mRNA, 2) 25 pg each of *egfp* and *bad,* 3) 25 pg each of *egfp* and *zbcl-xL*, or 4) 25 pg each of *bad* and *zbcl*-*xL*. At 24 hpf, half of each group was irradiated with 8 Gy IR and analyzed three hours later by the Casp3 assay. (B) Fluorescence intensity was measured in the spinal cords of at least 10 embryos from each group. Data represent one experiment, but the experiment was independently performed three times with similar results.

BAD’s pro-apoptotic function has been defined by its ability to bind and inactivate the anti-apoptotic Bcl-2 family members Bcl-2 and Bcl-xL to induce mitochondrial apoptosis [14]. However, Caspase 3 is activated by both the intrinsic mitochondrial apoptosis pathway and the extrinsic death receptor pathways [6], [7]. To confirm that BAD radiosensitizes embryos through activation of the mitochondrial pathway, we injected one-cell stage embryos with mRNA encoding *BAD* or *mcherry* in the presence or absence of *bcl-xL* mRNA, irradiated half of each group at 24 hpf and analyzed apoptosis three hours later by the Casp3 assay. Figure 5 shows that overexpression of *bcl-xL* almost completely inhibits BAD-mediated radiosensitization, indicating that Bad radiosensitizes zebrafish neural tissue by engaging the mitochondrial apoptosis pathway.

### Genetic Model of Bad-mediated Radiosensitization

**Figure 6:**
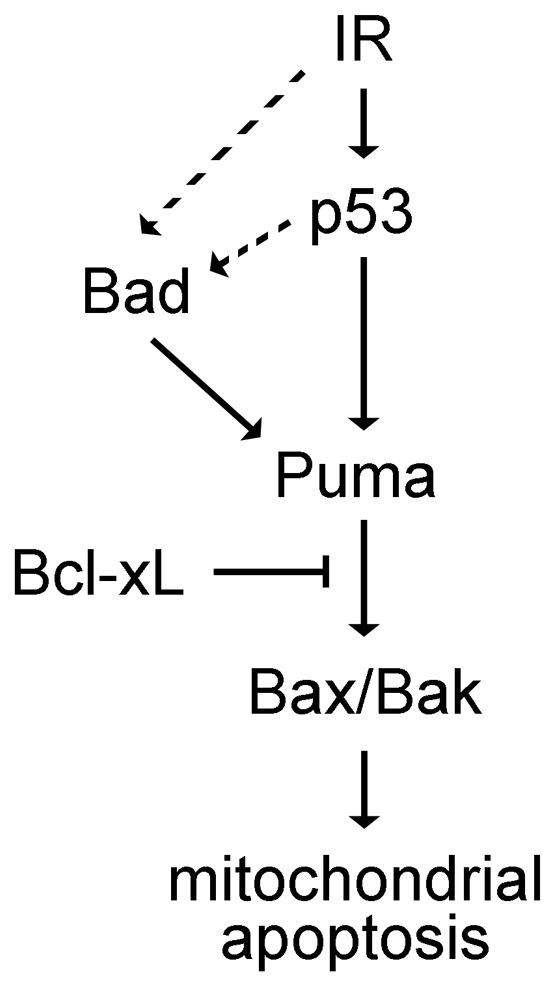
Genetic diagram of Bad-mediated radiosensitization of zebrafish neural tissue. IR activates the pro-apoptotic activity of Bad in a pathway that is either downstream of or parallel to p53 (dotted lines indicate that it is unclear whether this step occurs in a p53-dependent or –independent manner). Bad and Puma are dependent upon each other to promote IR-induced apoptosis. However, based on Bad’s established role as a sensitizer BH3-only protein and Puma’s reported role as an activator BH3-only protein, Bad likely functions upstream of Puma to induce IR-mediated apoptosis through the mitochondrial pathway.

Our data led us to devise a model (Figure 6) whereby IR leads to activation of Bad in a pathway that is either parallel to or downstream of p53 activity. While both Puma and Bad cause activation of Bax and Bak, only Puma has been shown to do so through direct interaction [39]. Our experiments demonstrating that Bad functions genetically upstream of Puma in response to IR suggest a hierarchy among these two BH3-only proteins whereby Bad functions as an essential sensitizer and Puma as an essential activator of IR-induced mitochondrial apoptosis.

## Discussion

A number of studies have interrogated the mechanism by which the various BH3-only proteins induce activation of BAX and BAK [5]. Early studies using peptides derived from individual BH3 domains suggested that both PUMA and BAD act indirectly to activate BAX and BAK, through binding to and inactivating anti-apoptotic Bcl-2 family proteins. PUMA and BAD were therefore considered “sensitizer,” “inactivator,” or “derepressor” BH3-only proteins [6], [8], [41]. However, it was subsequently shown using in vitro-translated full-length proteins that while BAD remained a *bona fide* sensitizer, PUMA is able to function as an activator [39]. These experiments further established the existence of a hierarchical relationship between sensitizer- and activator-BH3-only proteins in that sensitizers function genetically upstream of activators. In particular, it was shown that PUMA is required for BAD to induce apoptosis when overexpressed in mouse embryonic fibroblasts. Our genetic experiments establish that Bad and Puma also function in this hierarchical manner during IR-induced apoptosis in a whole animal system *in vivo*. Accordingly, we found that BAD requires Puma to induce apoptosis in response to IR (Figure 4). However, we unexpectedly found that Puma also requires Bad since loss of endogenous Bad almost completely inhibits IR-induced apoptosis (Figure 1). We and others have shown that overexpression of Puma alone quickly causes massive apoptosis followed by embryonic death [12], [13]. Our findings therefore suggest that the levels of Puma induced by p53 following exposure of zebrafish embryos to IR are not sufficient to overwhelm the endogenously expressed anti-apoptotic Bcl-2 family proteins and that Bad is likely to be necessary to release Puma from these proteins to directly activate Bax and Bak. Thus, Bad and Puma represent interdependent, critical components of IR-induced apoptosis in this system.

It is interesting that the neural tissue of the 24-hpf embryo is uniquely sensitive to Bad-mediated radiosensitization. Indeed, developing neurons are known to be exquisitely sensitive to DNA damage-induced apoptosis as mutations in critical mediators of the DDR, like ATM, cause neurodegenerative diseases [42]. One possible explanation lies in the observation that while *p53* is expressed in most tissues of the 24-hour zebrafish embryo (http://zfin.org/cgi-bin/webdriver?MIval=aa-pubview2.apg&OID=ZDB-PUB-040907-1), IR-induced *puma* induction appears to be concentrated in neural tissue [34]. Thus, the requirement for *puma* expression may explain why IR-induced apoptosis, as well as Bad-mediated radiosensitization, is restricted to neural tissue.

Since Bad is normally expressed in most tissues of the 24-hpf zebrafish embryo (http://zfin.org/cgi-bin/webdriver?MIval=aa-pubview2.apg&OID=ZDB-PUB-051025-1), and Bad overexpression induces only minimal, if any, apoptosis ([13] and Figures 5 and S1), Bad is likely to exist in a phosphorylated state of inactivation in developing zebrafish embryos. Following IR, Bad becomes pro-apopototic, likely through IR-induced serine dephosphorylation [13]. Thus, IR-mediated signaling probably acts to either inhibit the function of the kinases that target Bad, to induce activity of the phosphatases that desphosphorylate Bad, or both. Indeed, IR-induced activation of ATM has been shown to phosphorylate several proteins that could impact the phosphorylation state of Bad, including AKT, P70S6K, and the PP2A regulatory subunit PPP2R5D [43]. We have found that knockdown of *ppp2r5d* inhibits IR-induced apoptosis in zebrafish embryos (data not shown). Thus, it is plausible that Atm-mediated phosphorylation of Ppp2r5d contributes to the activation of Bad. Since Bad requires p53-mediated transcriptional activity to radiosensitize embryos (Figure 2A-B), it’s also possible that p53 regulates the expression of a kinase and/or phosphatase that targets Bad. Thus, the IR-induced activation of Bad could occur in a pathway that is either parallel to, or downstream from, p53 (Figure 6).

Bad is unique among the BH3-only proteins because its overexpression is compatible with normal development during early embryogenesis [12], [13]. Following the induction of DNA-DSBs by IR, Bad is transformed into a potent inducer of apoptosis. This could be advantageous from a therapeutic point-of-view since drugs that increase the expression of Bad (either transcriptionally or through protein stabilization) could potentially sensitize p53-wild-type cancer cells to IR. Future experimental studies could test this idea by examining the sensitivity of p53 wild-type tumors that ectopically express Bad to IR-induced ablation.

## Supporting Information

Figure S1 The ability of Bad to radiosensitize zebrafish embryonic neural tissue is conserved between zebrafish and human. Shown are lateral views of representative tails from 27-hpf wild-type embryos injected with 50 pg of mRNA encoding *mcherry* (cntl) or *hBAD*. Apoptosis in irradiated embryos is denoted with arrowheads. At 24 hpf, half of each group was irradiated with 8 Gy IR and analyzed three hours later by the Casp3 assay. Fluorescence intensity was measured in the spinal cords of at least 10 embryos from each group. Data represent one experiment, but the experiment was independently performed three times with similar results.
(TIF)

Figure S2 Diagram of *zbad* genetic knockdown strategies. (A) The *zbad b* gene (located on zebrafish chromosome 7) contains 4 exons (rectangles) and three introns (adjoining lines between the exons) with start (ATG) and stop (TGA) codons marked in the second and fourth exons, respectively. Primers *zbad-for* and *zbad-rev* were designed to amplify the complete coding sequence of the zebrafish *bad* gene. The *bad e2i2* MO was designed to inhibit the splice donor site at the junction of exon 2 and intron 2. The BH3 domain, which is required for Bad pro-apoptotic activity, is encoded at the end of exon 3. (B) One-cell stage embryos were injected with 200 nmol of either mismatch (mm) or *bad e2i2* MO. At 24 hpf, RNA was harvested from each group and analyzed by RT-PCR using either *bad-for* plus *bad-rev* primers, or primers that amplify *β-actin* as a loading control. (C) All four bands from the upper agarose gel pictured in (B) were excised and cloned into pGEM-T-easy for subsequent sequencing. The transcript resulting from each band is diagrammed based on sequencing results. The *bad e2i2* MO causes both inclusion of intron 2 and an in-frame deletion in exon 2, as well as production of low levels the wild-type *bad* transcript. Expected translation products are shown below the observed transcripts. The Δ18 deletion (represented by the 413-bp band in (B)) is expected to include the BH3 domain and could possibly give rise to a functional Bad protein. These results show that the *bad e2i2* MO induces an on-target, albeit incomplete, knockdown of the *zbad* gene.
(TIF)

Figure S3 Wild-type Bad synergizes with wortmannin to induced embryonic death. Shown are brightfield views of representative wells from a 12-well plate demonstrating morphological changes observed in the experiment quantified in Figure 2D. WM; wortmannin.
(TIF)

Figure S4 Bad-mediated radiosensitization does not alter the timing of IR-induced apoptosis. One-cell stage embryos were injected with 50 pg mRNA encoding *mcherry* (cntl) or *hBAD*. At 24 hpf, half of each group was irradiated with 8 Gy IR and analyzed one, two, and three hours later by the Casp3 assay. Fluorescence intensity was measured in the spinal cords of at least 10 embryos from each group, and the fluorescence intensity in control-injected embryos at 3 hpIR was normalized to 1.
(TIF)

Table S1 Sequences of primers and morpholinos used in the experiments described in this study.
(DOCX)
