# Supplementary figures and images for: Interdependence of Bad and Puma during Ionizing-Radiation-Induced Apoptosis
Source: PLoS One. 2014 Feb 6;9(2):e88151. doi: 10.1371/journal.pone.0088151 (PMC3916415; doi:10.1371/journal.pone.0088151)

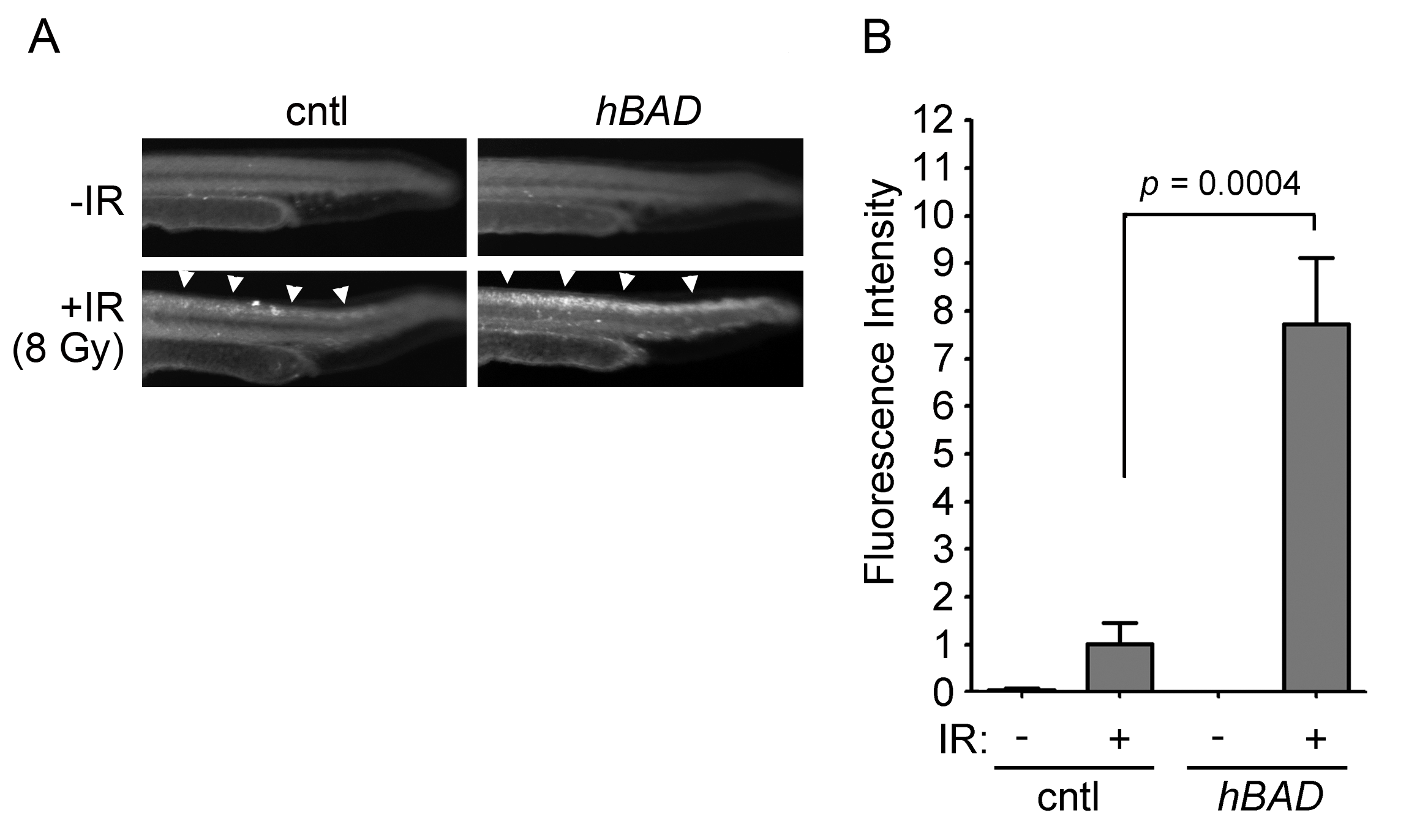

Supplement: Figure S1 — The ability of Bad to radiosensitize zebrafish embryonic neural tissue is conserved between zebrafish and human. Shown are lateral views of representative tails from 27-hpf wild-type embryos injected with 50 pg of mRNA encoding mcherry (cntl) or hBAD. Apoptosis in irradiated embryos is denoted with arrowheads. At 24 hpf, half of each group was irradiated with 8 Gy IR and analyzed three hours later by the Casp3 assay. Fluorescence intensity was measured in the spinal cords of at least 10 embryos from each group. Data represent one experiment, but the experiment was independently performed three times with similar results. (TIF) [file pone.0088151.s001.tif]

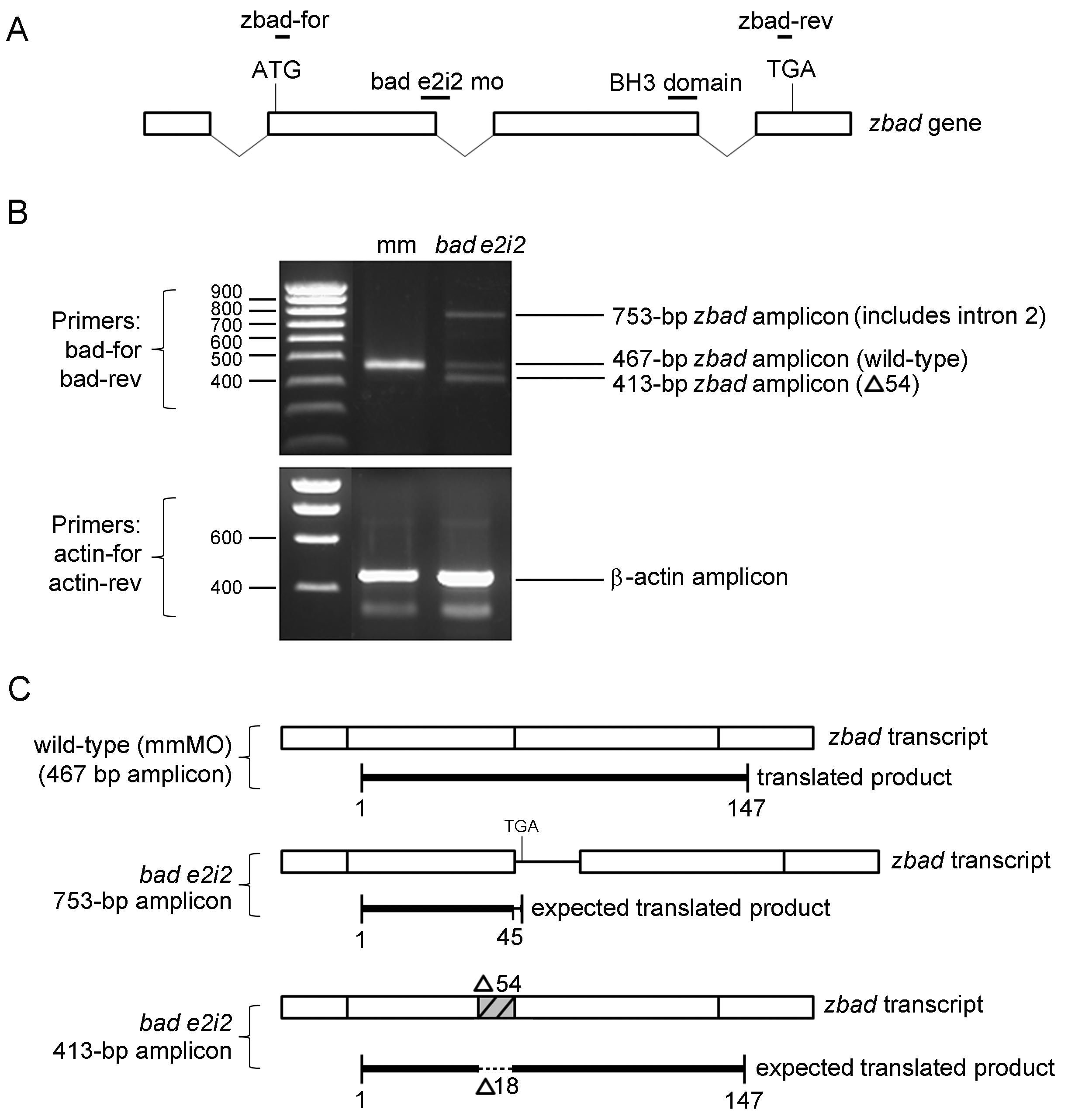

Supplement: Figure S2 — Diagram of zbad genetic knockdown strategies. (A) The zbad b gene (located on zebrafish chromosome 7) contains 4 exons (rectangles) and three introns (adjoining lines between the exons) with start (ATG) and stop (TGA) codons marked in the second and fourth exons, respectively. Primers zbad-for and zbad-rev were designed to amplify the complete coding sequence of the zebrafish bad gene. The bad e2i2 MO was designed to inhibit the splice donor site at the junction of exon 2 and intron 2. The BH3 domain, which is required for Bad pro-apoptotic activity, is encoded at the end of exon 3. (B) One-cell stage embryos were injected with 200 nmol of either mismatch (mm) or bad e2i2 MO. At 24 hpf, RNA was harvested from each group and analyzed by RT-PCR using either bad-for plus bad-rev primers, or primers that amplify β-actin as a loading control. (C) All four bands from the upper agarose gel pictured in (B) were excised and cloned into pGEM-T-easy for subsequent sequencing. The transcript resulting from each band is diagrammed based on sequencing results. The bad e2i2 MO causes both inclusion of intron 2 and an in-frame deletion in exon 2, as well as production of low levels the wild-type bad transcript. Expected translation products are shown below the observed transcripts. The Δ18 deletion (represented by the 413-bp band in (B)) is expected to include the BH3 domain and could possibly give rise to a functional Bad protein. These results show that the bad e2i2 MO induces an on-target, albeit incomplete, knockdown of the zbad gene. (TIF) [file pone.0088151.s002.tif]

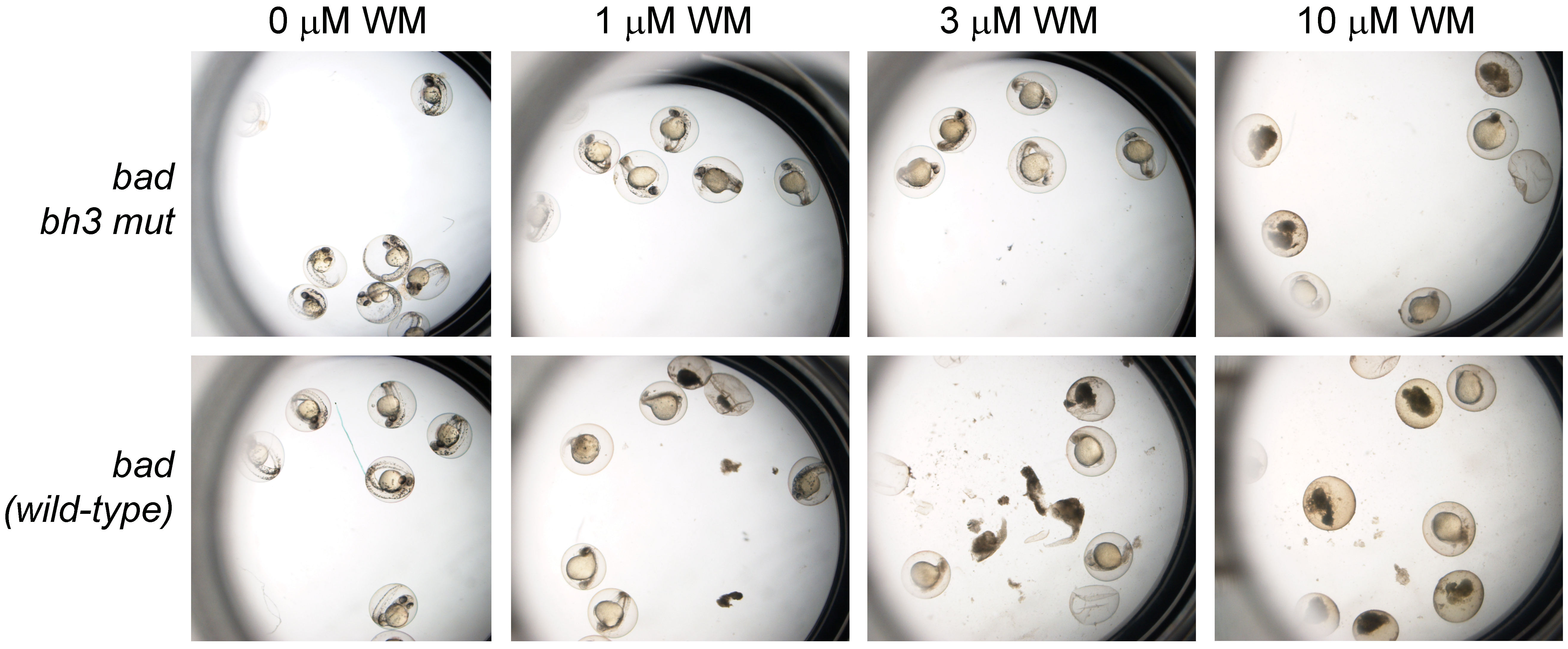

Supplement: Figure S3 — Wild-type Bad synergizes with wortmannin to induced embryonic death. Shown are brightfield views of representative wells from a 12-well plate demonstrating morphological changes observed in the experiment quantified in Figure 2D. WM; wortmannin. (TIF) [file pone.0088151.s003.tif]

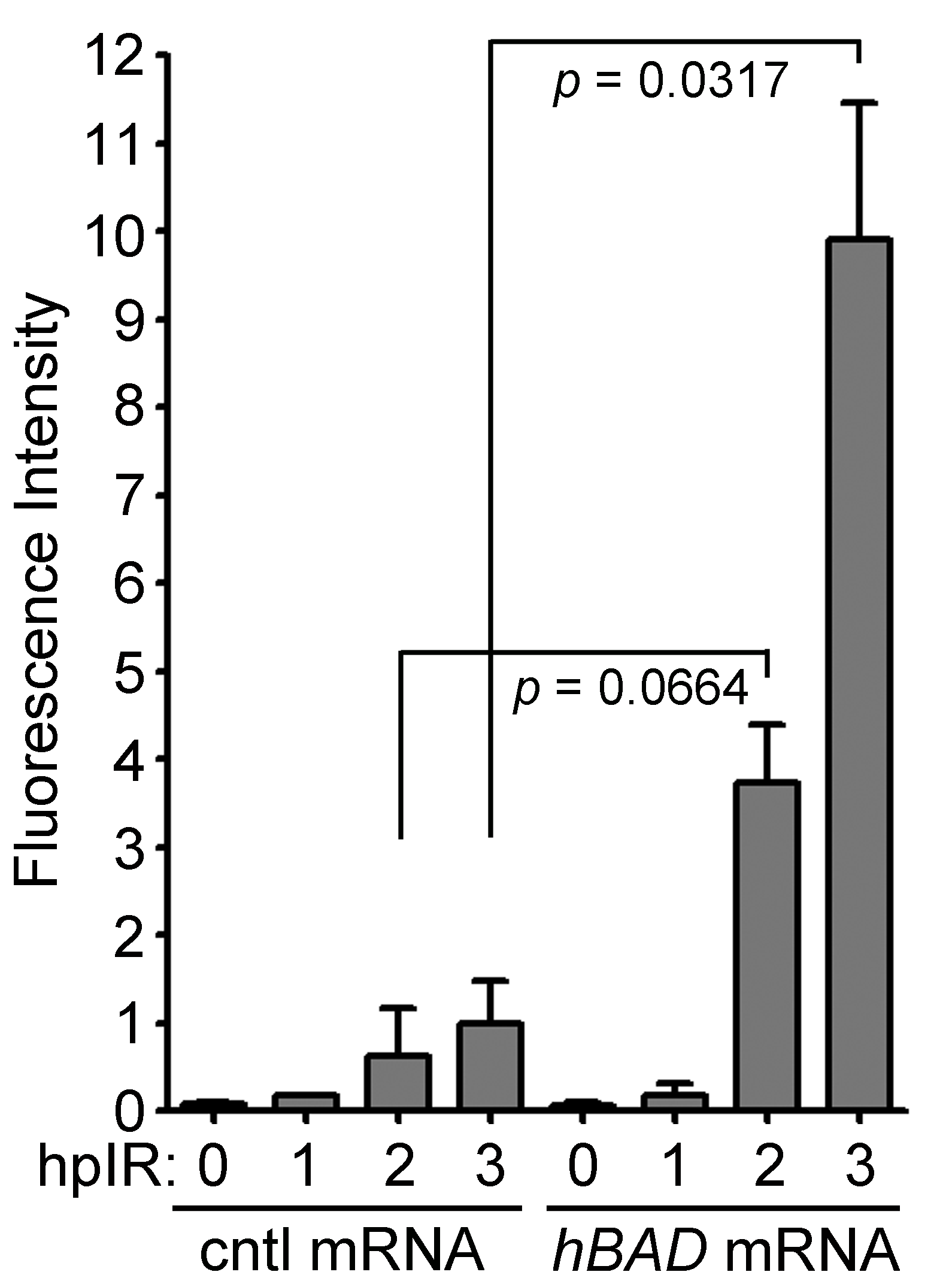

Supplement: Figure S4 — Bad-mediated radiosensitization does not alter the timing of IR-induced apoptosis. One-cell stage embryos were injected with 50 pg mRNA encoding mcherry (cntl) or hBAD. At 24 hpf, half of each group was irradiated with 8 Gy IR and analyzed one, two, and three hours later by the Casp3 assay. Fluorescence intensity was measured in the spinal cords of at least 10 embryos from each group, and the fluorescence intensity in control-injected embryos at 3 hpIR was normalized to 1. (TIF) [file pone.0088151.s004.tif]
