# Supplementary material for: Interdependence of Bad and Puma during Ionizing-Radiation-Induced Apoptosis
Source: PLoS One. 2014 Feb 6;9(2):e88151. doi: 10.1371/journal.pone.0088151 (PMC3916415; doi:10.1371/journal.pone.0088151)
Supplement: Supplementary file 2 [file pone.0088151.s005.docx]

Supplemental Experimental Procedures:

**(A) mRNA expression constructs: Primers for sub-cloning into pCS2+**	
pCS2-mcherry	
cherryCLAforCJ	CATGATCGATATGGTGAGCAAGGGCGAG
cherryR1revCJ	CATGGAATTCCTTGTACAGCTCGTCCATG
pCS2-zbad	
bad-for	CATGGAATTCATGGCACATATGTTTAATATCTC
bad-rev	CATGCTCGAGTCACTCTGCGGGGCGCG
pCS2-hBAD	
hBADforCJ	CATGGAATTCATGTTCCAGATCCCAGAGTTT
hBADrevCJ	CATGCTCGAGTCACTGGGAGGGGGCGG
pCS2-zbcl-xL	
Zfblp1-FOR-CJ	CATGGAATTCCATGTCTTACTATAACCGAGAAC
Zfblp1 – REV – CJ	CATGCTCGAGTCACAGGCGTTTCTGTGC
**(B) Morpholinos**	
mismatch	ATtTGTcCCATcATTcAACGTgTTG
translation-blocking *bad ATG*	ATATGTGCCATGATTGAACGTCTTG
splice-blocking *bad e2i2*	AGCCCTTGAGACTCACTCACCTTTC
splice-blocking *puma e2i2*	AACCAAGCATGACTCTTACCCTCTG
**(C) Primers for RT-PCR (validation of bad e2i2 morpholino specificity)**	
bad-for	CATGGAATTCATGGCACATATGTTTAATATCTC
bad-rev	CATGCTCGAGTCACTCTGCGGGGCGCG
zb-actinRTforCJ	CTGGTCGTTGACAACGGCT
zb-actinRTrevCJ	TCCATCACAATACCAGTAGT
**(D) zPCR primers and probes (Roche Universal Probe Library)**	
zpuma	
zpuma probe:	Universal Probe #18
zpuma-for:	GCCTTCAGCTTGGACAGC
zpuma-rev:	GGACACTTCCTGTTCTGTTCCT
zp21	
zp21 probe:	Universal Probe #142
QRTp21F	AAGCGCAAACAGACCAACAT
QRTp21R	TCAGCTACTGGCCGGATTT
zgapdh	
zgapdh probe:	Universal Probe #147
zgapdh-for:	CAGGCATAATGGTTAAAGTTGGTA
zgapdh-rev:	CATGTAATCAAGGTCAATGAATGG
